# Mid-Infrared Polarization Spectroscopy Measurements of Species Concentrations and Temperature in a Low-Pressure Flame

**DOI:** 10.1177/0003702818823239

**Published:** 2019-03-27

**Authors:** Anna-Lena Sahlberg, Dina Hot, Rasmus Lyngbye-Pedersen, Jianfeng Zhou, Marcus Aldén, Zhongshan Li

**Affiliations:** Division of Combustion Physics, Lund University, Lund, Sweden

**Keywords:** Mid-infrared polarization spectroscopy, low pressure flame, dimethyl ether, methane, quantitative concentrations, temperature

## Abstract

We demonstrate quantitative measurements of methane (CH_4_) mole fractions in a low-pressure fuel-rich premixed dimethyl ether/oxygen/argon flat flame (Φ = 1.87, 37 mbar) using mid-infrared (IR) polarization spectroscopy (IRPS). Non-intrusive in situ detection of CH_4_, acetylene (C_2_H_2_), and ethane (C_2_H_6_) in the flame was realized by probing the fundamental asymmetric C–H stretching vibration bands in the respective molecules in the spectral range 2970–3340 cm^−1^. The flame was stabilized on a McKenna-type porous plug burner hosted in a low-pressure chamber. The temperature at different heights above the burner (HAB) was measured from the line ratio of temperature-sensitive H_2_O spectral lines recorded using IRPS. Quantitative measurements of CH_4_ mole fractions at different HAB in the flame were realized by a calibration measurement in a low-pressure gas flow of N_2_ with a small admixture of known amount of CH_4_. A comprehensive study of the collision effects on the IRPS signal was performed in order to quantify the flame measurement. The concentration and temperature measurements were found to agree reasonably well with simulations using Chemkin. These measurements prove the potential of IRPS as a sensitive, non-intrusive, in situ technique in low pressure flames.

## Introduction

Low-pressure laminar flames have been widely employed for the investigation of the combustion chemistry of different fuels.^1^ The distribution of different combustion intermediate species, as the key information, is commonly measured with probing techniques, such as molecular beam mass spectroscopy (MBMS).^2–4^

Dimethyl ether (DME) has been widely studied as a potential alternative fuel to diesel and biodiesel.^4–7^ A detailed study of DME–O_2_–Ar flames at several different flame conditions using MBMS was performed by Wang et al.^3^ While MBMS is useful for studying many species at the same time, it is also an intrusive probing technique, which may affect the flame chemistry.^8,9^ It is therefore of crucial importance to develop non-intrusive optical techniques for concentration measurements in low pressure flames.

Polarization spectroscopy (PS) was first demonstrated by Wieman and Hänsch^10^ in 1976 as a spatially resolved Doppler-free laser technique. Since then, it has been widely applied for combustion diagnostics. The PS technique combines many of the merits of laser diagnostics, such as high spatial resolution, in situ, species-selective, and non-intrusive detection. Polarization spectroscopy has been applied in combustion environments to detect for example OH,^11–13^ C_2_,^14^ and NO.^15^

Infrared PS (IRPS) is a sensitive, nonlinear laser technique that is useful for studies of molecular species that lack easily accessible electronic transitions. Specifically, many important hydrocarbon species can be studied through the asymmetric C–H vibration bands around 3 µm. Infrared PS has been applied in combustion research to detect, for example, H_2_O,^16,17^ CO_2_,^16^ CH_4_,^18,19^ and HCl,^20^ and has been applied in low-pressure flames for measurements of OH,^17^ as well as CH_4_ and C_2_H_2_.^21^ Sun et al. demonstrated quantitative measurements of C_2_H_2_^22^ and HCN^23^ concentrations in atmospheric pressure flames using IRPS. Flame temperature measurements using the line ratio of hot H_2_O lines around 3.1 µm measured by a similar technique, IR degenerate four-wave mixing (IR-DFWM), was demonstrated in atmospheric pressure flames.^24^

This paper investigates IRPS as a tool for both temperature and quantitative concentration measurements of combustion intermediates in low-pressure flames. The flame studied here is a rich DME/O_2_/Ar flame (Φ = 1.87) at 37 mbar. The flame temperature was measured from the recorded line ratio of hot H_2_O lines in the flame, using a method similar to the one presented by Sun et al.^24^ but with a direct calibration. Spectral lines from CH_4_, C_2_H_2_, and C_2_H_6_ were identified in the IRPS excitation scans through comparison with simulations using molecular parameters extracted from the HITRAN^25^ and HITEMP^26^ databases. Quantitative CH_4_ mole fractions were calculated from the IRPS signal at different heights above the burner (HAB) using a calibration measurement in a low-pressure N_2_ gas flow carrying trace amounts of CH_4_ of known concentration. The dependence of the IRPS signal on temperature, pressure, and buffer gas environment was investigated in order to quantify the flame measurements. The measured temperature and CH_4_ mole fractions were compared with simulations from Chemkin^27^ using the reaction mechanism for DME presented by Zhao et al.;^28^ the measurements agree well with the simulations within the limits of measurement uncertainties.

## Theory

The theory of PS has been extensively presented.^29–31^ In a PS experiment, a tunable laser beam is split into a weak probe beam and a strong pump beam which are crossed in the detection volume. The probe beam is linearly polarized, while the pump beam is either circularly polarized, or linearly polarized at a 45° angle to the probe beam polarization. The probe beam is aligned through two crossed polarizers, which effectively block the probe beam. The pump beam is aligned to cross the probe beam between the polarizers. When the laser is in resonance with an absorption line of a selected molecular species in the measurement volume, the absorption of the strong pump beam introduces a birefringence for the probe beam in the detected gases. The polarization of the linearly polarized probe beam will thus change when passing through the sample, which means part of the probe beam can pass through the second polarizer, forming the PS signal.

An empirical method for quantitative concentration measurements using IRPS has been described.^22,23^ Assuming an optically thin sample, saturating laser energies, and ignoring the leakage of laser light through the crossed polarizers, the line-integrated IRPS signal *I* can be written as

(1) $$I\propto N_{0}^{2}\sigma^{2}I_{\mathrm{laser}}\zeta_{{\mathrm{JJ}}^{'}}^{2}\mathrm{gc}$$

where *N*_0_ is the ground state population of the molecules, σ is the absorption cross-section of the transition defined for a single molecule, *I_laser_* is the laser intensity, *g* is a parameter accounting for the spectral overlap between laser profile and the absorption profile of the molecular line, and *c* is a parameter accounting for the collision effects under different conditions (temperatures, pressures, and buffer gases). The variable *g* accounts for the absorption linewidth, including both the Doppler broadening and the collision broadening. ζ*_JJ_′* is a geometry factor of the pumped transition, which depends on the pump beam polarization and the rotational quantum number *J* of the upper and lower states. A linearly polarized pump beam will enhance the Q-branch lines of the probed molecular species while suppressing the P- and R-branch lines, and a circularly polarized pump beam will enhance the P- and R-branch lines, while suppressing the Q-branch lines.^29,30^

The mole fraction *f*_2_ of a species in a flame can be calculated from the IRPS signal as^22,23^

(2) $$f_{2}=f_{1}\cdot \frac{T_{2}}{T_{1}}\cdot \frac{\sigma_{1}}{\sigma_{2}}\cdot (\frac{g_{1}}{g_{2}}{)}^{\frac{1}{2}}\cdot (\frac{c_{1}}{c_{2}}{)}^{\frac{1}{2}}\cdot (\frac{I_{2}}{I_{1}}{)}^{\frac{1}{2}}$$

where *f*_1_ is the mole fraction in the calibration measurement, σ is the absorption cross-section from the HITRAN database, and *T* is the temperature. The subscripts indicate the parameter for the (1) calibration measurement and the (2) flame measurement, respectively. The factors *g*_1_/*g*_2_ and *c*_1_/*c*_2_ can be determined from a calibration of the signal dependence on temperature.

The experimental equipment used in these measurements did not have a heating device inside the low-pressure chamber. Therefore, IRPS signals at high temperatures were recorded at atmospheric pressure. Assuming the same gas composition, the collision broadening of a spectral line with pressure and temperature can be approximated as^32,33^

(3) $${\Delta \nu }_{\mathrm{coll}}(p,T)={\Delta \nu }_{\mathrm{coll}}(p_{0},T_{0})\cdot \frac{P}{P_{0}}\cdot (\frac{T_{0}}{T}{)}^{0.7}$$

where Δν*_coll_*(*p*_0_,*T*_0_) is the collision broadening at the reference temperature *T*_0_ and pressure *p*_0_. Based on this, it was assumed that the pressure and temperature effects on the spectral linewidth of the IRPS signal could be considered separately.

The measurements in this work were performed using saturating laser intensities since the empirical model for concentration measurements assumes this. Even though the linewidth of the absorption lines can be affected by the laser saturation, non-saturating IRPS signals are more sensitive to collisions.^34^

## Experimental

### Infrared Polarization Spectroscopy Setup

The laser system used in these experiments has been described in detail by Li et al.^18^ and only a brief description is presented here. The second harmonic of an injection-seeded neodymium-doped yttrium aluminum garnet (Nd:YAG) laser (Spectra Physics, PRO 290-10) was used to pump a dye laser (Sirah, PRSC-D-18), giving tunable laser light around 800 nm. The dye laser beam was then frequency mixed in a LiNbO_3_ crystal with part of the residual 1064 nm output from the Nd:YAG laser. This beam was further amplified in another LiNbO_3_ crystal, to provide mid-IR laser tunable radiation from 2900 cm^−1^ to ∼3400 cm^−1^ with pulse energies around 4–5 mJ and a pulse duration of ∼4 ns. The linewidth of the final mid-IR output has been measured to be 0.025 cm^−1^.^16^

**Figure 1.**
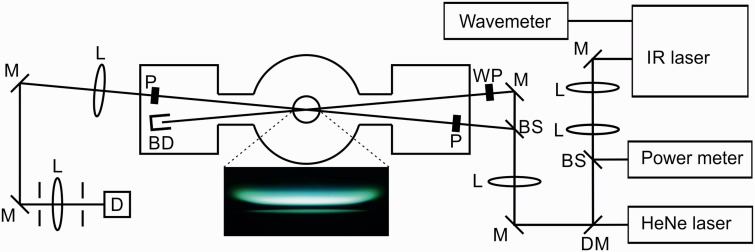
Schematic of the experimental setup. M: mirror, L: CaF_2_ lens, BS: CaF_2_ window beam splitter, DM: dichroic mirror, WP: λ/4 or λ/2 waveplate, P: YVO_4_ Glan laser polarizer, BD: beam dump, D: InSb detector. The photograph shows the DME/O_2_/Ar flame (Φ = 1.87) at 37 mbar.

Figure 1 shows a schematic of the experimental setup. This experiment used co-propagating pump and probe beams. A telescope was used to collimate the mid-IR laser beam. The reflection from a CaF_2_ window was sent to a power meter to monitor the laser energy during the scan. To facilitate alignment, the mid-IR beam was overlapped with a HeNe laser beam. Part of the residual dye laser beam after the frequency mixing was directed to a wavemeter (HighFinesse, WS/6 High Precision–UV), to monitor the laser wavelength during the scans.

The probe beam was generated by a 7% reflection from a CaF_2_ window; the rest of the laser beam was used as a pump beam. The pump beam was passed through either a λ/4 or a λ/2 waveplate to generate a circularly or linearly polarized pump beam. For probing the R-branch lines in CH_4_ and C_2_H_2_, a circularly polarized pump beam was used, while a linearly polarized pump beam was used for probing the Q-branch lines in C_2_H_6_. The pump and probe beams were crossed with ∼4° angle in the center of the low-pressure flame. An *f* = 750 mm CaF_2_ lens was used to focus the pump and probe beams over the burner. The size of the interaction region was estimated to be 0.5 × 0.5 × 10 mm^3^. A better resolution along the length of the laser beams can be achieved. However, there is a tradeoff between the spatial resolution and the signal strength since the signal is proportional to the square of the interaction length.^29^ The vertical position of the burner could be adjusted in order to probe different height above the burner (HAB) in the flame. Two YVO_4_ IR polarizers aligned at crossed angles were placed in the probe beam path before and after the flame. To avoid interference from birefringence in the windows of the chamber, the polarizers were placed inside the low-pressure chamber, in two specially designed side chambers.^21^ After the burner, the pump beam was directed to a beam dump. The probe beam passed through the second polarizer and was collimated with an *f* = 1000 mm CaF_2_ lens and directed to the liquid nitrogen cooled InSb detector (Judson technologies, J10D-M204-R04M-60).

The flow speeds of the gases to the chamber were controlled using mass flow controllers (Bronkhorst). Nitrogen was flushed into the side chambers to prevent water vapor from condensing on the windows and the surfaces of the polarizers.

### Flame

The flame used in these experiments was a rich low-pressure DME/O_2_/Ar flame stabilized on a McKenna-type porous plug burner of 6 cm in diameter. The equivalence ratio, Φ, of the flame was 1.87 and the DME/O_2_/Ar mole fractions were set to 0.286/0.459/0.255. The cold gas flow speed was 62.9 cm/s and the pressure was kept at 37 mbar. The flame composition was simulated with Chemkin^27^ using the reaction mechanism for dimethyl ether developed by Zhao et al.^28^

## Measurements

### Temperature

Temperature is one of the most important parameters in combustion. Therefore, many laser techniques have been developed for non-intrusive, in situ measurements of flame temperatures, including coherent anti-Stokes Raman scattering,^35,36^ laser-induced fluorescence,^37,38^ two-line atomic fluorescence,^33,39^ laser-induced grating spectroscopy,^40,41^ and degenerate four-wave mixing.^42,43^ However, an accurate and precise measurement of flame temperature in low-pressure flames is still a challenging task.

Sun et al. proposed a measurement technique where the line ratio of two hot H_2_O lines in the mid-IR spectral region, measured using IR-DFWM, was used to evaluate the flame temperature.^24^ The H_2_O spectral lines were chosen because they possessed a lower state energy difference sensitive to flame temperature changes, were relatively free from spectral interference, and had negligible line strength at room temperature, which is important to avoid interference from laser absorption in the ambient air. The same method was adopted here to measure the flame temperature at different HAB in the flame using the line ratio of hot H_2_O lines recorded with IRPS.

**Figure 2.**
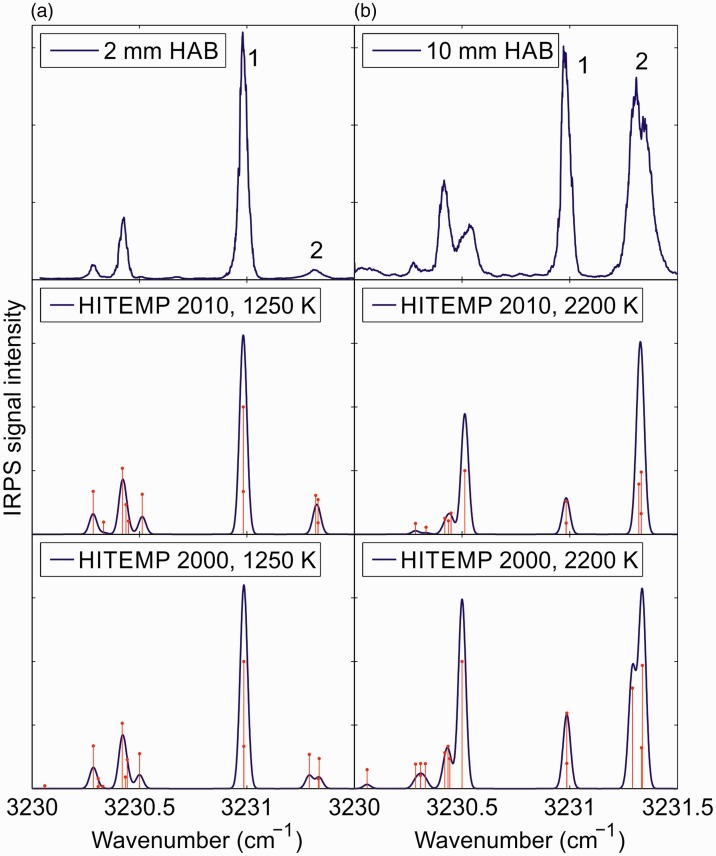
Infrared PS excitation scan of H_2_O lines in the flame at (a) 2 mm HAB and (b) 10 mm HAB. Each scan is an average of 10 consecutive scans. The scans are compared with simulations of the IRPS signal at 1250 or 2200 K using data from the HITEMP 2010 and HITEMP 2000 databases. The red bars in the simulations indicate the separate transitions in the simulation data. Line data for the transitions are shown in Table I.

Figure 2 shows IRPS excitation scans over three H_2_O line groups at 2 mm and 10 mm HAB in the flame, respectively. Each scan is an average of 10 consecutive measurements. The IRPS intensity ratio of the line at (1) 3230.98 cm^−1^ to the line at (2) 3231.33 cm^−1^ varies dramatically at the different HAB, which shows the potential for sensitive measurements of the temperature in the flame. The absorption lines are weak enough that re-absorption of the signal in the flame is <10%, which will have a negligible effect on the line ratio.

Also shown in Fig. 2 are simulations of the IRPS signal of H_2_O using data from HITEMP 2010 database^26^ and from the HITEMP 2000 database. As was shown by Sun et al.,^24^ the HITEMP 2000 database seems to better reproduce the measured IRPS signals of lines 1 and 2, in terms of the linewidth of line 2. The line data for the transitions in lines 1 and 2 in the HITEMP 2010 and 2000 databases are summarized in Table I.

**Table I.** Line data for the H_2_O transitions for lines 1 and 2 at 1000 K from HITEMP 2010^a^ and 2000.^b^

Line	Wavenumber (cm^−1^)	Line intensity, 296 K $(\frac{{\text{cm}}^{-1}}{\text{molec}\cdot {\text{cm}}^{-2}})$	Lower state energy (cm^−1^)	Upper vibrational level	Lower vibrational level	Upper rotational level	Lower rotational level
1	3230.9830^a^ 3230.9861^b^	1.672 × 10^−21^ 7.770 × 10^−22^	1789.0428 1789.0410	1 0 0	0 0 0	7 7 1	8 8 0
1	3230.9833^a^ 3230.9861^b^	4.970 × 10^−21^ 2.331 × 10^−21^	1789.0428 1789.0410	1 0 0	0 0 0	7 7 0	8 8 1
2	3231.3206^a^ 3231.2905^b^	5.866 × 10^−22^ 1.150 × 10^−21^	5035.1265 5035.1040	0 0 1	0 0 0	17 8 10	18 8 11
2	3231.3316^a^ 3231.3328^b^	1.423 × 10^−22^ 3.807 × 10^−22^	5713.2500 5713.3230	0 0 1	0 0 0	23 0 23	24 0 24
2	3231.3316^a^ 3231.3368^b^	4.277 × 10^−22^ 1.145 × 10^−21^	5713.2500 5713.3230	0 0 1	0 0 0	23 1 23	24 1 24

As seen in Fig. 2, there are substantial differences between the measured and simulated H_2_O spectrum in this spectral region. Like DFWM, simulations of the IRPS signal of closely spaced transitions are very sensitive to the accurate line positions of the involved transitions.^34,44,45^ Uncertainty in the accuracy of the line positions of the transitions in HITEMP could be the reason for the difference between the measurement and the simulations. Simulating a DFWM or PS spectrum is a complex task, since overlapping transitions affect each other.^34,46^ If the positions of closely spaced lines are not accurate, the simulated spectrum can look very different from the measured.

In order to calibrate the H_2_O line ratio to flame temperatures, IRPS measurements of the H_2_O lines were performed in the product zone of low-pressure CH_4_/O_2_/N_2_ flames with Φ = 1.16, 1.42, and 1.68 at 50 mbar. The temperature in the same flames has been measured by Borggren et al.^33^ using the two-line atomic fluorescence (TLAF) technique. Recording the line ratio in these flames gives a calibration for the H_2_O line ratio at different temperatures. The flame conditions and the calibration data are shown in Table II.

**Table II.** Flame conditions for the CH_4_/O_2_/N_2_ flames used for the calibration of the H_2_O line ratio versus temperature.

Φ	CH_4_/O_2_/N_2_ mole fractions	Flame temperature at 7 mm HAB^33^	Ratio of the line-integrated IRPS signal for H_2_O line 1 to line 2
1.16	0.207/0.351/0.442	2150	0.66
1.42	0.261/0.365/0.374	2350	0.43
1.68	0.378/0.454/0.168	2550	0.35

**Figure 3.**
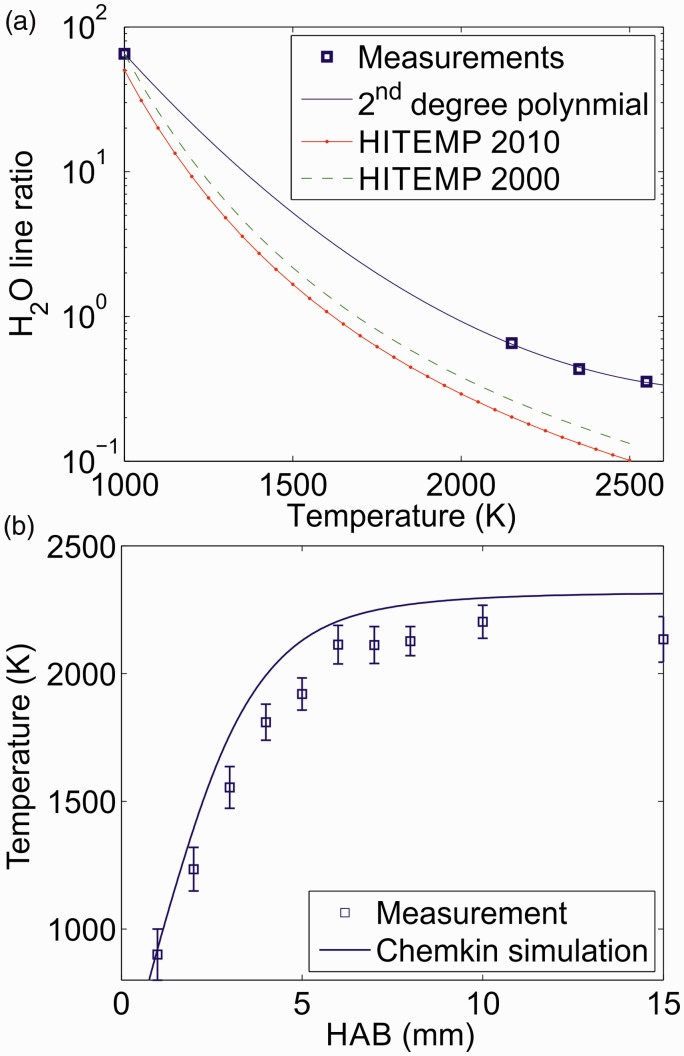
(a) Calibration curve of the line-integrated IRPS signal ratio of the H_2_O lines, compared with simulations of the line ratio using data from HITEMP 2010 and 2000. (b) The temperature at different HAB measured by the H_2_O line ratio (squares) and simulated from Chemkin (solid line). The error bars are the standard deviation of 10 separate measurements.

Figure 3a shows the calibration curve for the line-integrated H_2_O line ratio versus temperature. The squares show the calibration points of the line ratio versus temperature measured in the low-pressure CH_4_/O_2_/N_2_ flames. The point at 1000 K was taken from the simulation from HITEMP 2000. The blue line shows a polynomial fit to the calibration points, which was used to extrapolate the calibration curve between 1000 and 2600 K. The red curve shows the simulated line ratio using data from HITEMP 2010 and the green curve shows the line ratio from HITEMP 2000. It is clear that if the simulated line ratio is used, the flame temperature can be underestimated by > 500 K.

Figure 3b shows the temperature estimated from the measured H_2_O line ratio at different HAB. The line ratio was estimated by integrating the IRPS signal over the line profile and calculating the ratio of the integrated signal of the two lines. Using the blue calibration curve in Fig. 3a, the line ratio can then be matched to a temperature value. Each measurement point in Fig. 3b is an average of the line ratio estimated from 10 different measurements at the same HAB; the error bars show the standard deviation of the estimated temperatures from each measurement. A total uncertainty of ±100 K was estimated for the evaluated temperature. Part of this comes from the standard deviation of the temperature from different scans, which is around ±50 K. Another part of this stems from the uncertainty in the simulated line ratio at 1000 K and in the accuracy of the calibration points. Including a 50% uncertainty in the simulated ratio gives the total uncertainty of ±100 K for the temperature measurement.

The measurements are compared with a Chemkin simulation of the adiabatic flame temperature. The simulated and measured temperature values agree reasonably well. The lower measured flame temperature is probably due to heat losses in the flame to the burner and the ambient air, which was not accounted for in the simulation. Similar temperature differences have been shown before.^47,48^

### Detection of Intermediate Hydrocarbon Species

**Figure 4.**
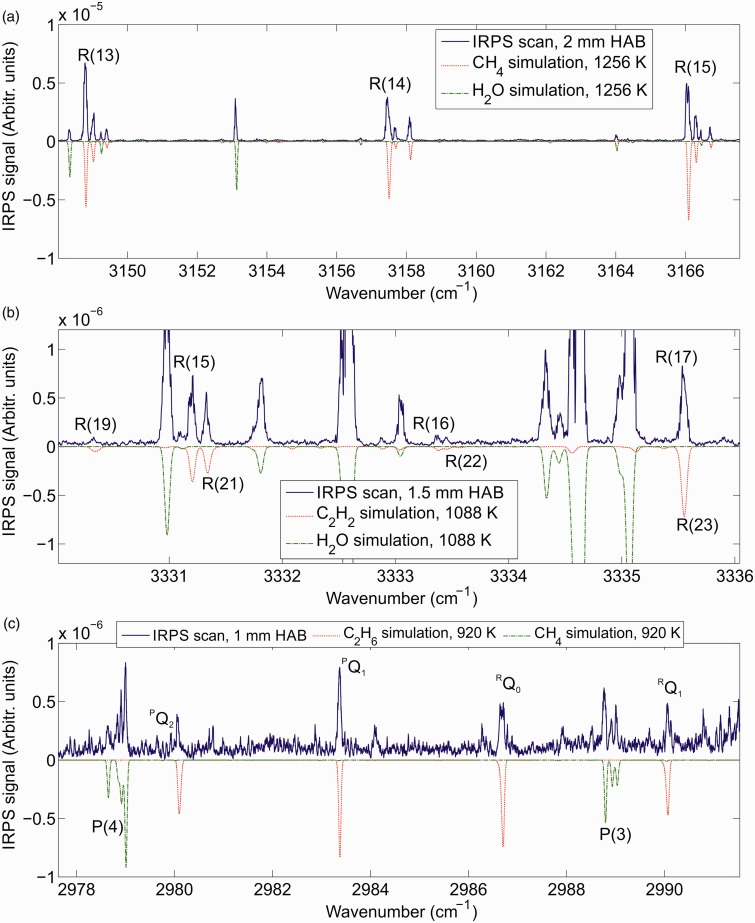
Infrared PS excitation scans in different wavelength intervals at (a) 2 mm HAB, (b) 1.5 mm HAB, and (c) 1 mm HAB. Comparison with simulations using data from the HITRAN and HITEMP databases allows for identification of CH_4_, C_2_H_2_, and C_2_H_6_ lines in the measured spectra.

Infrared PS has previously been applied for species detection in low-pressure flames.^17,21^ Shown in Fig. 4 are IRPS excitation scans recorded at different HAB in the flame and in different spectral regions. Simulations of the IRPS signal based on the molecular data extracted from the HITRAN and HITEMP databases are used to identify spectral lines in the measured spectra. The simulations are calculated according to Eq. 1, but without accounting for collisions, spectral overlap, or laser intensity fluctuations. The relative intensities of the simulations were adjusted to fit the measurements. It should be noted that the data from HITRAN are not always complete at high temperatures.^49–52^

Figure 4a shows an IRPS excitation scan at 2 mm HAB. By comparing the measurement with the simulation, the *R*(13)*–R*(15) lines of the ν_3_ band of CH_4_ can be identified, along with several H_2_O lines. Due to the symmetry fine structure, each R-branch line is split into several lines.^53–55^

Figure 4b shows an IRPS excitation scan at 1.5 mm HAB. In this spectral region, several C_2_H_2_ lines are identified including the *R*(15)*–R*(17) lines from the ν_3_ band, the *R*(21)*–R*(23) lines from the ν_2_ + (ν_4_ + ν_5_)^0^ band, and the *R*(19) line of the ν_2_ + ν_4_^1^–ν_4_^1^ hot band of C_2_H_2_. The *y*-axis scale in the figure is zoomed in to highlight the weaker C_2_H_2_ lines in the spectrum. The strong water lines in the scan are thus saturated in the figure.

Figure 4c shows an IRPS excitation scan at 1 mm HAB. In this spectrum, a linearly polarized pump beam was used in order to enhance the Q-branch lines in the C_2_H_6_ spectrum. The ^P^Q_2_–^R^Q_1_ lines of the *v*_7_ band of C_2_H_6_ are identified in the spectrum by comparison with the simulation. The HITRAN database line list for C_2_H_6_ is not complete in this spectral region, containing only data for the high intensity Q-branches of the ν_7_ band.^25^ The absorption spectra of C_2_H_6_ in this spectral range has been investigated at room temperature^56^ and elevated temperatures,^49,51,52^ showing the existence of several P- and R-branch transitions between the Q-branches. These transitions can probably account for many of the unidentified lines in the IRPS spectrum in Fig. 4c. The spectrum also contains signals from the P(4) and P(3) CH_4_ lines. Using a linearly polarized pump beam suppresses the strength of P- and R-branch lines. The CH_4_ lines are weak in this spectrum compared to Fig. 4a and there are no H_2_O lines visible in this spectral range.

From these signal intensities and the calculations of concentrations using CHEMKIN, we estimate a detection limit in the flame of 1.1 × 10^16^ molecules/cm^3^ (1900 ppm) for CH_4_, 2.7 × 10^15^ molecules/cm^3^ (400 ppm) for C_2_H_2_, and 7.6 × 10^15^ molecules/cm^3^ (950 ppm) for C_2_H_6_.

### Quantitative Methane Concentration Measurement

In this paper, the R(13) line of the ν_3_ band of CH_4_ was used for quantitative measurements of the CH_4_ mole fraction in the flame. CH_4_ is an intermediate species which is formed and consumed in the flame zone of the DME/O_2_/Ar flame. The R(13) line was chosen for having negligible interference from other species in the flame and also having a reasonably high absorption cross-section both in the flame and at room temperature. The R(13) line consists of several transitions due to the symmetry fine structure.^53–55^ Table III shows the line data from the HITRAN database for the seven transitions forming the first peak at 3148.8 cm^−1^ within the R(13) line. Line notations from HITRAN are used for the vibrational and rotational levels.

**Table III.** Line notations for the symmetry fine structure transitions of the seven transitions forming the symmetry fine structure peak at 3148.8 cm^−1^ of the R(13) line of the ν*_3_* band of CH_4_.

Wavenumber (cm^−1^)	Line intensity, 296 K $(\frac{{\text{cm}}^{-1}}{\text{molec}\cdot {\text{cm}}^{-2}})$	Lower state energy (cm^−1^)	Upper vibrational level		Lower vibrational level		Upper rotational level		Lower rotational level	
3148.7839	1.017 × 10^−20^	950.3850	0 0 1 0	1F2'	0 0 0 0	1A1'	14A1	17	13A2	1
3148.8110	4.597 × 10^−21^	950.5222	0 0 1 0	1F2'	0 0 0 0	1A1'	14F2	52	13F1	4
3148.8135	3.747 × 10^−21^	950.5049	0 0 1 0	1F2'	0 0 0 0	1A1'	14E	34	13E	2
3148.8214	2.685 × 10^−21^	950.3048	0 0 1 0	1F2'	0 0 0 0	1A1'	14F2	49	13F1	3
3148.8237	5.224 × 10^−21^	950.3368	0 0 1 0	1F2'	0 0 0 0	1A1'	14F1	47	13F2	2
3148.8299	5.522 × 10^−21^	950.4872	0 0 1 0	1F2'	0 0 0 0	1A1'	14F1	49	13F2	3
3148.8581	2.744 × 10^−21^	950.3048	0 0 1 0	1F2'	0 0 0 0	1A1'	14F2	50	13F1	3

The mole fraction *f*_2_ of a species in the flame can be determined from Eq. 2, by comparison with a calibration measurement performed at room temperature in a gas flow with known concentration.^22,23^ To relate the calibration measurement to the flame measurement, the changes in collision factors *c* and spectral overlap factor *g* with temperature have to be investigated. In order to study this, the full width at half maximum (FWHM) of the R(13) line was measured at several different conditions and the results were used to estimate the factors *c* and *g*.

First, the IRPS signal change with temperature was investigated in atmospheric pressure gas flows of N_2_ with small admixtures of CH_4_. The flows were heated in a T-shaped glass tube surrounded by electric heating wire. The concentration of CH_4_ in the N_2_ gas flow was kept at ∼1000 ppm. The scan was performed over the R(13) line of the ν_3_ band of CH_4_.

**Figure 5.**
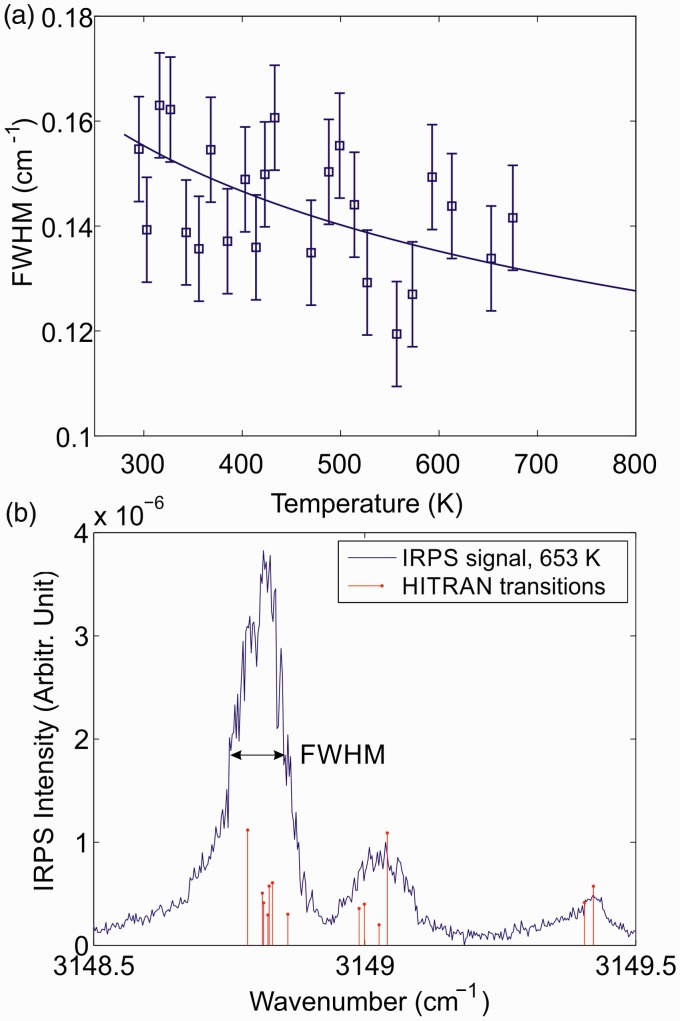
(a) The FWHM of the IRPS signal of the R(13) line of CH_4_ as a function of temperature. The measurements were performed in atmospheric pressure gas flows of N_2_ with ∼1000 ppm of CH_4_ admixed. The solid line is a polynomial fit to the measurements. (b) Infrared PS excitation scan of the R(13) line of CH_4_ at 653 K. The FWHM is defined in the figure. The red bars show the relative line strength at 653 K of the individual transitions taken from the HITRAN database.

Figure 5a shows the spectral linewidth of the IRPS signal of the CH_4_ R(13) line recorded at different temperatures. Figure 5b shows the IRPS signal recorded at 653 K. The symmetry fine structure is partially resolved in the scan. The red bars show the relative line strength at 653 K of the individual transitions, taken from the HITRAN database. The linewidth displayed in Fig. 5a was defined as the FWHM for the peak at 3148.8 cm^−1^, as illustrated in Fig. 5b. Due to the unstable mode structure of the multimode pump laser, the line shape of the scanned CH_4_ line becomes uneven and the shape varies between different scans. This is the reason for the relatively large spread of the measured FWHM versus temperature. Using a single-mode laser source would greatly improve the stability of the FWHM measurement.^57^ As the temperature increases, the lines get narrower since the collision rate in the gas decreases. The uncertainty of the measured FWHM was estimated to be ±0.01 cm^−1^, which is illustrated by the error bars. The solid line is a polynomial fit to the measurements. As the measured line consists of several transitions from the symmetry fine structure, the FWHM is larger than for a single line and the temperature dependence on the linewidth does not exactly follow the predicted behavior from Eq. 3.

**Figure 6.**
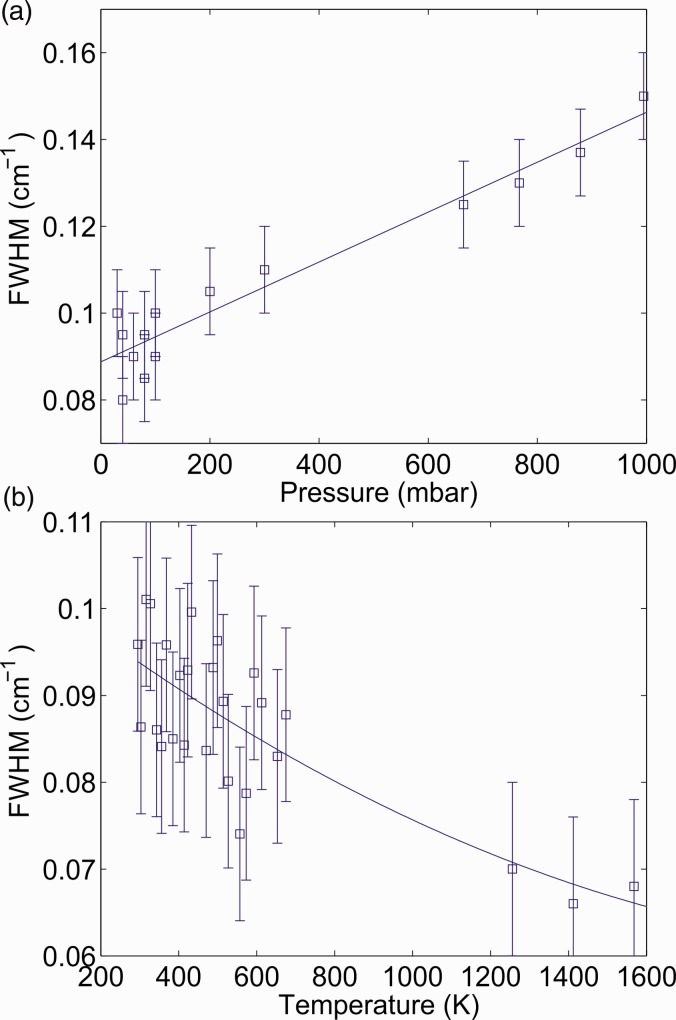
(a) The FWHM of the IRPS signal of the R(13) line of CH_4_ as a function of pressure. The solid line is a linear polynomial fit to the measurements. (b) The FWHM of the IRPS signal at 37 mbar as a function of temperature, estimated from the temperature dependence in Fig. 5a and the pressure dependence in (a). The three points above 1200 K are the FWHM retrieved from IRPS signals of the R(13) line at different HAB in the low-pressure flame. The solid line is a polynomial fit to the measurements.

Next, the pressure dependence of the IRPS signal of the R(13) line at room temperature was investigated. Figure 6a shows the FWHM of the recorded IRPS signal as a function of pressure. The solid line is a linear polynomial fit to the measurements, showing that the FWHM at 37 mbar is ∼38% lower than at atmospheric pressure. Since the R(13) line consists of several closely spaced transitions, the pressure dependence is more complicated than the simple relationship described in Eq. 3. However, the pressure dependence can still be well described with a linear fit.

According to Eq. 3, the pressure and temperature effects on the collision width of a spectral line can be considered as separate effects. This means that the temperature dependence of the FWHM at 37 mbar should follow the same curve as the one at atmospheric pressure displayed in Fig. 5a, except with a lower FWHM due to the lower pressure, as shown in Fig. 6a. Figure 6b shows the estimated FWHM of the IRPS signal at 37 mbar as a function of temperature. The FWHM of the R(13) line at three HAB in the flame were also included to complete the analysis. The temperature for these points is taken from the temperature measurement in Fig. 3 and the values have a previously stated uncertainty of ±100 K. At flame temperatures, the FWHM of the signal seems to be constant within the measurement uncertainty.

To account for the Doppler broadening of the R(13) line of CH_4_, the Doppler width can be calculated as ${\Delta \nu }_{D}=\nu_{0}\cdot \sqrt{8\ln(2)\mathrm{kT}/{\mathrm{mc}}^{2}}$, where ν_0_ is 3148.8 cm^−1^, *k* is the Boltzmann constant, *T* is the temperature, *m* is the mass of a CH_4_ molecule, and *c* is the speed of light in vacuum. The laser line shape is characterized by a Gaussian with FWHM of 0.025 cm^−1^ and the collisional linewidth at different temperatures was estimated from Fig. 6b. The IRPS signal intensity was then simulated at room temperature and flame temperature by convoluting the laser line profile with the estimated molecular absorption line profile. From these calculations, we estimate the ratio of the spectral overlap factor *g* in the calibration gas and in the flame to be *g*_1_/*g*_2_≈0.88 at 37 mbar.

**Figure 7.**
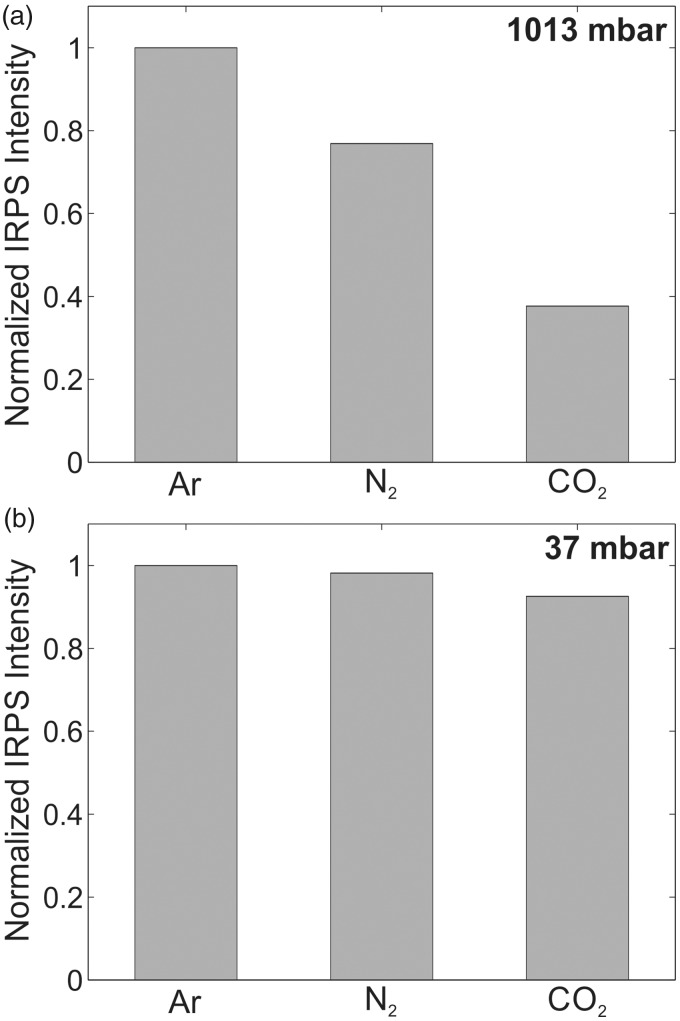
The relative line-integrated IRPS signal of the CH_4_ R(13) line in the different buffer gases at (a) atmospheric pressure and (b) 37 mbar. The signals are normalized to the measurement in Ar.

At low pressures, the IRPS signal is expected to be less sensitive to the collision environment, due to much lower collision rate between the molecules in the gas.^21,58,59^ To test this, the IRPS signal for the R(13) line of CH_4_ was recorded in three different buffer gases (Ar, N_2_, and CO_2_) at atmospheric pressure and at 37 mbar. Figure 7 shows the line-integrated IRPS signal in the different buffer gases at atmospheric pressure (Fig. 7a) and 37 mbar (Fig. 7b). The concentration of CH_4_ in the gas flows was 2200 ppm at atmospheric pressure and 15 000 ppm at 37 mbar.

It is clear from Fig. 7 that the IRPS signal intensity is only weakly dependent on the collision environment at 37 mbar. At atmospheric pressure, the difference in the signal intensity for signals recorded in buffer gas argon and N_2_ is 23%, and between argon and CO_2_ the difference is 62%. However, at 37 mbar, signals in N_2_ and CO_2_ buffer gas only differ 1.8% and 7.4%, respectively, compared to the signals in Ar buffer gas. The average time between collisions in an ideal gas can be calculated from the temperature, pressure, the mean molecular weight, and the mean diameter of the molecules in the gas.^60^ Table IV shows the calculated average time between collisions in a N_2_ gas at different temperatures, both at atmospheric pressure and at 37 mbar. Based on the fact that the average collision time is comparable to or even longer than the laser pulse duration, we assume the signal dependence on changes in the collision environment due to different temperature will be negligible at 37 mbar, and that there is only a small difference in the collisional quenching between a low-pressure gas flow and a low-pressure flame. Therefore, we estimate the ratio of the collisional quenching factor *c* in the calibration gas and in the flame to be *c*_1_/*c*_2_≈1 at 37 mbar. An uncertainty of 10% was estimated for the factors *g*_1_/*g*_2_ and *c*_1_/*c*_2_.

**Table IV.** The average time between collisions in a N_2_ gas mixture at different temperatures and pressures.

Temperature (K)	296	1000	1500	2000
1013 mbar	0.14 ns	0.25 ns	0.31 ns	0.35 ns
37 mbar	3.7 ns	6.8 ns	8.4 ns	9.6 ns

**Figure 8.**
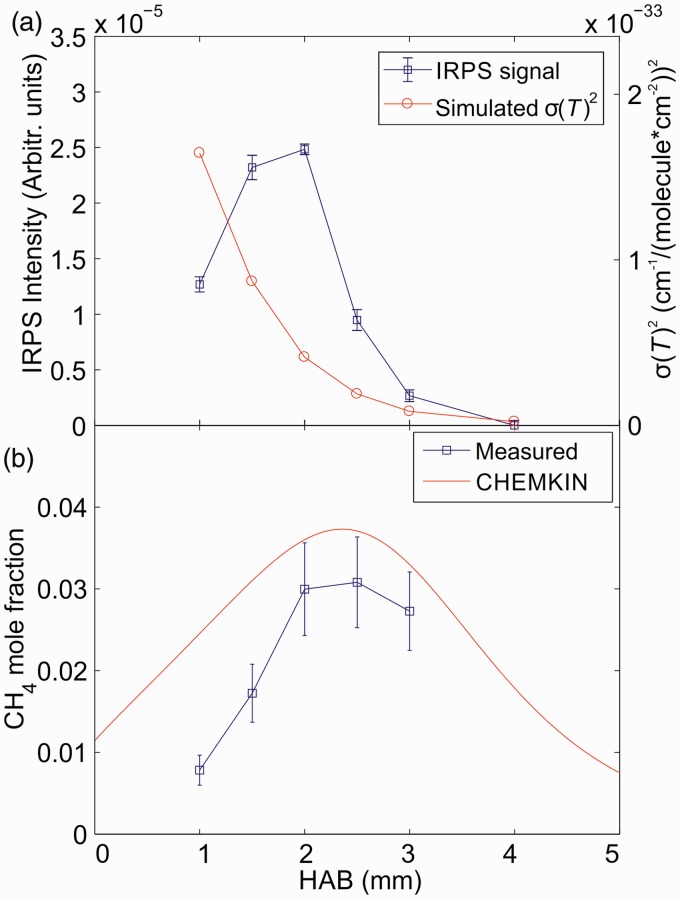
(a) The measured IRPS line-integrated signal intensity at each HAB, compared with the square of the simulated absorption cross-section (σ(*T*)^2^) at each HAB. (b) The measured and simulated CH_4_ mole fraction as a function of the HAB in the flame. The error bars reflect the uncertainty in temperature, *g*_1_/*g*_2_ and *c*_1_/*c*_2_ factors, and the IRPS signal uncertainty.

The line-integrated IRPS signal for the R(13) line in CH_4_ was recorded at different HAB. The signal is shown in Fig. 8a together with the square of the simulated absorption cross-section (σ(*T*)^2^) at each HAB, which was extracted from the HITRAN database. The temperature from Fig. 3b was used in the calculation of σ(*T*). To perform quantitative CH_4_ concentration measurements, the IRPS signal was recorded in a 37 mbar N_2_ calibration flow with 12 400 ppm CH_4_ admixed. The mole fraction of CH_4_ in the flame was then calculated using Eq. 2. The values for the *g*_1_/*g*_2_ and *c*_1_/*c*_2_ factors were taken as estimated earlier in the calculation.

Figure 8b shows the measured CH_4_ mole fraction versus HAB compared with the simulation from Chemkin. The error bars are a combination of the estimated uncertainty in temperature, absorption cross-section, *g*_1_/*g*_2_ and *c*_1_/*c*_2_ factors, and the standard deviation of the IRPS signal. As has been shown before,^22,23^ the largest contribution to the error bars comes from the temperature uncertainty since the temperature is also used to calculate the absorption cross-section. The measured mole fraction corresponds reasonably well with the simulation within the measurement uncertainty. At lower HAB, the difference between the measurement and simulation increases. Further studies are needed to determine the cause of this difference.

## Conclusion

We have demonstrated the application of IRPS as a spatially precise spectroscopic technique for temperature measurements, species detection, and quantitative species concentration measurements in low-pressure flames. Spectral lines from C_2_H_2_, CH_4_, and C_2_H_6_ were identified in IRPS excitation scans by comparison with the simulations using data from the HITRAN database. The measurements illustrate the potential of IRPS for non-intrusive detection of combustion intermediate species in low-pressure laminar flames.

Quantitative measurements of CH_4_ mole fractions at different HAB in the flame were measured using an on-line calibration method.^22,23^ The IRPS signal dependence on temperature, pressure, and buffer gas environment was investigated in order to improve the accuracy of the calibration. Temperature measurements in the flame at different HAB were performed using the relative line-integrated IRPS signal ratio of two H_2_O lines. The line ratio of the measured H_2_O lines has previously been shown to be very sensitive to temperature changes in the range of 1000–2000 K.^24^ The technique shows great promise for non-intrusive temperature measurements in low-pressure flames. The measured temperature was used for simulations of the absorption cross-section of the CH_4_ R(13) line using data from HITRAN. By recording the IRPS signal of the R(13) line of CH_4_ diluted in different buffer gases (N_2_, Ar, and CO_2_), it was found that the IRPS signal is relatively insensitive to changes in the collision environment at sub-atmospheric pressure (37 mbar) compared to atmospheric pressure. The factors *g*_1_/*g*_2_ and *c*_1_/*c*_2_ that correct for the changes in spectral overlap and collision between the room temperature calibration flow and the flame measurements were found to be almost negligible at 37 mbar. This indicates a good potential for accurate quantitative measurements of concentrations of species in low-pressure flames.

In conclusion, this work shows the potential of IRPS for sensitive detection of hydrocarbon species in laminar flames and for quantitative measurements of temperatures and species mole fractions. Besides flame diagnostics, this technique could also be utilized for diagnostics in biomass gasification, catalysis, and other harsh environments. The main uncertainty in the measured mole fractions comes from the temperature uncertainty, since that plays a role in the simulated absorption cross-section as well as for the temperature dependence of the signal. For the temperature measurements with IRPS, future work is needed to acquire a more accurate calibration for the line ratio versus temperature and to investigate how this changes under different pressures. Using a single-mode laser could greatly improve the IRPS signal stability, which would improve the precision of the line ratio and the mole fraction measurements.^57^
